# Supplementary material for: Older people’s challenges and expectations of healthcare in Ghana: A qualitative study
Source: PLoS One. 2021 Jan 19;16(1):e0245451. doi: 10.1371/journal.pone.0245451 (PMC7815149; doi:10.1371/journal.pone.0245451)
Supplement: S1 File — (DOCX) [file pone.0245451.s001.docx]

**Audit Trail for aged care research**

19^th^ December 2018

The team had a meeting and discussed some interview techniques that are supposed to be used in collecting data. Ethical clearance was received from Kwame Nkrumah University of Science and Technology (KNUST) ethical review board. Pre-test of interview guide discussed.

20^th^ December 2018

The interview guide was modified by the team for more clarity. Issues of trustworthiness was also discussed at this meeting.

29^th^ December 2018

Data collection at Santasi area in the Ashanti Region. Five older people in the community were interviewed with the interview guide. The older people expressed their challenges and expectations with healthcare when they visit the hospital in the region. The main challenges that were expressed included issues with waiting time and having difficulty in locating departments in the hospital without any form of assistance. They expected that government will employ more nurses to reduce their waiting time and also show them places in the facilities. All older people said there should be special places in the hospital where they could be cared for. Some of the older persons compared situation to pregnant care in the hospital and said they should also have a similar system. Data transcribed same day.

30^th^ December 2018

Team planned on taking more data today. Five older people were in interviewed at Patasi area in the Ashanti region. The older persons talked about their frustrations when they get to hospitals in the Ashanti region with inadequate information on how things happen around the hospital and what they should do. Frustrations expressed also included having to pay so much money even though they went to the hospital with health insurance cards. Other older people also complained about lack of information to them at the outpatient department and other units of the hospitals that they visited. Older people were of the view the cost should be reduced for them and more attention given to their needs than it is currently and thought there should be frequent check up on them. Data was transcribed same day. Very similar issues were described today just like yesterday.

1^st^ January 2019

Team meeting to discuss some issues related to interviews and find out some of the main issues being identified in interviews

2^nd^ January 2019

Meeting with prof to tell her how the interviews are going and to plan interviews in the Southern part.

3^rd^ January 2019

Interviews were conducted with five older persons in the southern part of Ghana. They described a number of challenges they faced in the hospital regarding nursing care and also some expectations. The challenges included lack of information from nurses and other staff in terms of getting around the hospital and also delays they experienced in the hospital. Expectations mentioned were mainly subsidization in cost, having units or wards dedicated to them and nurses checking them up regularly in the community. Data was transcribed same day.

4^th^ January 2019

Interview with 5 more older people to find out their challenges and expectations. Participants talked about the need to have more information from nurses when they visit the hospital. They did not expect to ask before they are told what they should do but should have enough explanations on how things are supposed to happen in the hospital. They said nurses should find a way to prevent them from queuing when they come to the hospital since it was very frustrating for them. Having to pay various sums of monies in the hospital was another issue that bothered participants. When asked what they thought could be done, they said there should be subsidy for them, so they don’t have to pay all the monies they are paying. They also wanted more attention regarding their care and frequent checkups They felt they should have their own nurses and doctors and units. Data transcribed same day.

6^th^ January 2019

Meeting of the research team to discuss issues regarding data collection. Prof was in attendance. Some findings were discussed at this meeting. Plans were made for interviews at the northern part.

10^th^ January 2019

Semi-structured interviews held with five older people in the Northern part. Challenges mentioned in these interviews included financial constraints faced when they visited the hospital, delays in the hospital, inadequate information in the hospital. Staying too long in the facility was a worry. Expectations mentioned were focusing nursing care on the needs of older people, checking older people up regularly just like what nurses do for pregnant women. Data was transcribed in the evening of the interview

1^st^ February 2019

Interviews with five older people in the Northern part of Ghana. Older people mentioned several challenges including issues of cost and national health insurance not working well because they still pay various sums of money in the hospital. Expectations mentioned included having dedicated units and consulting rooms. Very similar findings to yesterday was found. Participants did not want to queue for service and also have to look for places in the hospital without anyone giving them the direction.

3^rd^ February 2019

Meeting was held with the team to see themes that were coming up and whether there was a need to do more interviews. Prof was in attendance.

4th^th^ February 2019

Team met to start analysis of data collected

5^th^ February 2019

Analysis of data collected continued.

29^th^ February 2019

Themes developed were shown to some participants to see whether they agreed with them.

1^st^ March 2019

Team started writing up manuscript.

2^nd^ march 2019

A meeting was held regarding journal that manuscript should be sent to. The team finally agreed on Plos one.
