# Supplementary material for: Older people’s challenges and expectations of healthcare in Ghana: A qualitative study
Source: PLoS One. 2021 Jan 19;16(1):e0245451. doi: 10.1371/journal.pone.0245451 (PMC7815149; doi:10.1371/journal.pone.0245451)
Supplement: S2 File — (DOCX) [file pone.0245451.s002.docx]

Interview Data

Participant 1

I have several challenges ooo my son. You see we don’t have the information we need in the hospital here. Nurses don’t explain much to us but I think they should be doing that. And then you know people are always many in the hospital so you can’t even finish quickly and go home. I was there two weeks ago and I can tell you it was not easy. I regretted even going but I had no choice. You have to be in the line for a long time. It is not the best. On top of that you have us paying some monies I don’t even understand. It looks like the health insurance is not working. ……Why is that happening. Hmmm, we are old so we should not be paying all these monies like that. Ooo sometimes I wish we had our own units or departments. To be honest with you, when I get there, nurses just make me join all these long lines of people. Sometimes I feel pity for myself in such situations. Sometimes I just buy some local medicines and take to help myself rather than going to the hospital. But when the local medicines are not helping, I go to the hospital.

Participant 2

Hmmm, well we need information oooo. We can’t just be roaming around all the time like that in the hospital trying to find places on our own. Why can’t there be any assistance to us. People can show us where we need to go around to avoid all these mary go round thing. It takes a tow on me. Sometimes too you don’t really know what is expected of you in the hospital. Very difficult you know when you don’t have much information. Every time I go to the hospital here they make me go through a lot of frustrations. I pay a lot of money, and I am made to wait aaaa for a long time. I don’t like it at all. The nurses here should try and make things easier for us. The government can help them set up new units for us. It will help us. They can also check up on us in our homes. It will be vey good. They do for pregnant women so they can also do for us.

Participant 3

Nurses did not explain issues into details when taking care of me. Most of the time they will come and say, Maame I want to do this and that for you. Maybe it is because they don’t have time. Not much explanation is given on the needs and how that task will affect my health. We also need more information on how to prevent diseases from getting to us. I think more explanations will be good. I will be happy if that can be done. I will always feel very comfortable with that part when they take their time to explain issues to us. I really think it is the lack of time in the hospital and workload. It is not easy when you think of going to the hospital. All that comes to mind is the long queues that will be waiting for you when you get there. Sometimes I just go to the drug store or the local chemist shop to get something for myself instead. But I think it is not the best. Check-ups can be done through calls to us by nurses, so we know whether we really need to come to the hospital. I am reluctant to take a car all the way to the hospitals for check-up although I know check-ups are good for my health and I know it can prevent serious diseases. They can just call us and talk to us.

Participant 4

They don’t really explain issues into details when taking care of me. Sometimes nurses will come and say, I want to do this and that for you. Not a lot of explanation is given on the needs and how important that task will affect my health. Maybe information too on prevention will also be good. I think more explanations will be good. I will be happy if that can be done. I will always feel very comfortable with our nurses telling exactly what is going to happen. It may be the lack of time in the hospital and workload. It is not easy when you think of going to the hospital. All that comes to mind is the long queues that will be waiting for you when you get there. Sometimes I get some drugs from the drug store or the local chemist shop for myself instead. But it is not good. Can they check on us at our homes? I think it is possible. Check-ups can be done through calls to us by nurses, so we know whether we really need to come to the hospital. I am reluctant to take a car all the way to the hospitals for check-up although I know check-ups are good for my health and I know it can prevent serious diseases. They can just call us and talk to us.

Participant 5

There are issues when I go to the hospital. There is little information and a lot of wasting of my time. I always leave the hospital very late and also end up paying huge sums of monies for procedures which I think it’s unfair to me as an old person who had served this county well. I don’t like joining long lines to see nurses and doctors or before having any of my procedures. I get frustrated by it, seriously. They should just have a separate unit for us and may be our own staff to take care of us. You know I have to go the hospital several times and things don’t seem to change in terms of the things we have complained all the time about.

Participant 6

I don’t like coming here because of a number of reasons. The nurses keep me for too long. All that comes to mind when I am about to come to the hospital is the long queues that will be waiting for me when I get there. Sometimes I just get some drugs from the drug store or the local chemist shop to get something for myself instead. But it is not good. Can they check on us at our homes? The government and the workers here should try and do that for us. It is possible. We also deserve good treatment. Even in our homes, we are ready for them to come to us and treat us. It will save us a lot of trouble. Check-ups can be done through calls to us by nurses, so we know whether we really need to come to the hospital. I am reluctant to take a car all the way to the hospitals for check-up although I know check-ups are good for my health and I know it can prevent serious diseases. They can just call us and talk to us.
