# Supplementary material for: Older people’s challenges and expectations of healthcare in Ghana: A qualitative study
Source: PLoS One. 2021 Jan 19;16(1):e0245451. doi: 10.1371/journal.pone.0245451 (PMC7815149; doi:10.1371/journal.pone.0245451)
Supplement: S1 Appendix — (DOCX) [file pone.0245451.s003.docx]

Appendix 1

**INTERVIEW GUIDE**

This study is being conducted to find out challenges and expectation of older persons in Ghanaian hospitals. This interview guide contains open ended questions. You are expected to kindly provide genuine answers to the questions. The information you provide is confidential and will be used only for the purposes of this study. If you have any question, do not hesitate to ask the researchers. Your cooperation and participation until the completion of the interview is very necessary for the successful completion of the study. However, your participation in this study is entirely up to you. You will not be penalized in anyway by refusing to participate.

**SECTION A: socio-demographic data**

**Kindly tick as appropriate:**

1. Age of respondent.

a. 60-64 years

b. 65-69 years

c. 70-74 years

g. 75-79

h. 80-84

i. 85-89

j. 90-94

k. 95-99

l. other………………..

1. Educational status
2. No formal Education
3. Nursery/Kindergarten
4. Primary school
5. Junior high school/JHS
6. Senior high school/SSS
7. Tertiary
8. Occupation
9. Employed
10. nurse,
11. doctor,
12. police
13. Trader
14. Pastor
15. Seamstress/tailor
16. Driver
17. Farmer
18. Hair dresser
19. Food vender
20. Unemployed
21. House wife
22. Student
23. Retired
24. Others………………
25. Religion
26. Islam
27. Christianity
28. Traditional Religion
29. Any other religion……………..

5. Marital Status

a. Single

b. Co-habitation

c. Married

d. Divorced

e. Widowed

f. Separated

g. Any other……………

6. Number of Children

a. None

b. One

c. Two

d. Three

e. Four

f. other, state…………..

**INTERVIEW SECTION**

**SECTION B:** **challenges of older persons**

7. Have you visited district hospitals during the year? Yes……No………

8. Kindly describe the care you were given in the hospital.

8. Tell us about the various supports that you had when you visited the hospital for care.

9. How will you describe the nursing care provided you?

10. Can you explain any problems you had when you visited the hospital? What were the problems or challenges

11. Describe any bothering issues you want addressed in the hospital.

**SECTION C: expectations**

12. Describe any expectations you had when you visited the hospital

13. Were your expectations met. If yes why do you say so? If no why do you say so?

Explain.

13. Do you have any expectations for health workers that care for you when you visit the hospital? If yes can you describe these expectations? If no can you explain why?

14. Do you have any suggestions for the improvement of nursing care of older persons? If yes can you describe them? If no can you explain why?
