# Supplementary material for: Older people’s challenges and expectations of healthcare in Ghana: A qualitative study
Source: PLoS One. 2021 Jan 19;16(1):e0245451. doi: 10.1371/journal.pone.0245451 (PMC7815149; doi:10.1371/journal.pone.0245451)
Supplement: Attachment — Submitted filename: PONE-D-19-28368_reviewer.pdf [file pone.0245451.s005.pdf]

PLOS ONE

Older people's challenges and expectations of nursing care in Ghana: A qualitative Study.

--Manuscript Draft--

**Manuscript Number:**	PONE-D-19-28368
**Article Type:**	Research Article
**Full Title:**	Older people's challenges and expectations of nursing care in Ghana: A qualitative Study.
**Short Title:**	Older people's challenges and expectations of nursing care in Ghana.
**Corresponding Author:**	CONFIDENCE ALORSE ATAKRO, MN<br>Christian Service University College<br>Kumasi, Kumasi GHANA
**Keywords:**	Elderly, advocacy, expectations, challenges, nursing, district, hospital, Ghana
**Abstract:**	Background<br>Elderly persons across the globe are increasingly becoming marginalised and isolated. The population of elderly persons in Ghana is expected to increase from 4.92% in 1960 to 6.34% in 2020. The increase in the number of elderly persons has not had a corresponding increase in social and health care support systems for the elderly. There is limited evidence of the nursing care challenges and expectations by elderly persons in Ghana. This study aimed at exploring nursing care challenges and expectations of elderly persons to inform policy that could lead to improved quality of life for elderly persons in Ghana.<br><br>Materials and Methods<br>Qualitative exploratory descriptive study design was used in conducting this study. Semi-structured interviews were used in collecting data from 30 participants from three regions in Ghana (10 from each region). Data analysis was carried out through content analysis.<br><br>Results<br>Five themes were extracted from data. These themes were: 1.Inadequate information from nurses. 2. Queuing frustrations. 3. Financial burden. 4. A cry for cost subsidisation. 5. Focused elderly care demand.<br><br>Conclusion<br>The elderly in Ghana experience challenges of nursing care which include inadequate information, queuing frustrations and financial burdens. Elderly persons also have expectations of care which include subsidisation of cost and attention to their care needs by nurses. Many of these challenges and expectations cannot be addressed by nurses alone. There is a need for collaborative approach as well as advocacy, curricula modifications and workshops for practicing nurses on improved elderly care in Ghana.
**Order of Authors:**	CONFIDENCE ALORSE ATAKRO, MN
	Abigail Atakro
	Janet Sintim Aboagye
	Alice Aluwah Blay
	Stella Boatemaa Addo
	Dorcas Frempomaa Agyare
	Peter Adatara
	Kwaku Gyimah Amoa-Gyarteng
	Awube Menlah
	Isabella Garti

Powered by Editorial Manager® and ProduXion Manager® from Aries Systems Corporation

	George Sedinam Boni
	Osei Kwaku Berchie
	Isaac Kwadwo Ansong
**Additional Information:**	
**Question**	**Response**
**Financial Disclosure**<br><br>Enter a financial disclosure statement that describes the sources of funding for the work included in this submission. Review the submission guidelines for detailed requirements. View published research articles from *PLOS ONE* for specific examples.<br><br>This statement is required for submission and **will appear in the published article** if the submission is accepted. Please make sure it is accurate.<br><br>**Unfunded studies**<br>Enter: *The author(s) received no specific funding for this work.*<br><br>**Funded studies**<br>Enter a statement with the following details:<br>• Initials of the authors who received each award<br>• Grant numbers awarded to each author<br>• The full name of each funder<br>• URL of each funder website<br>• Did the sponsors or funders play any role in the study design, data collection and analysis, decision to publish, or preparation of the manuscript?<br>• **NO** - Include this sentence at the end of your statement: *The funders had no role in study design, data collection and analysis, decision to publish, or preparation of the manuscript.*<br>• **YES** - Specify the role(s) played.<br><br>* typeset	The authors received no funding for this work. All funds for the study was provided by the first author.
**Competing Interests**<br><br>Use the instructions below to enter a competing interest statement for this submission. On behalf of all authors, disclose any competing interests that could be perceived to bias this	The authors have declared that no competing interests exists.

work—acknowledging all financial support and any other relevant financial or non-financial competing interests.<br><br>This statement **will appear in the published article** if the submission is accepted. Please make sure it is accurate. View published research articles from *PLOS ONE* for specific examples.<br><br>**NO authors have competing interests**<br><br>Enter: *The authors have declared that no competing interests exist.*<br><br>**Authors with competing interests**<br><br>Enter competing interest details beginning with this statement:<br><br>*I have read the journal's policy and the authors of this manuscript have the following competing interests: [insert competing interests here]*<br><br>* typeset	
**Ethics Statement**<br><br>Enter an ethics statement for this submission. This statement is required if the study involved:<br><br>• Human participants<br>• Human specimens or tissue<br>• Vertebrate animals or cephalopods<br>• Vertebrate embryos or tissues<br>• Field research<br><br>Write "N/A" if the submission does not require an ethics statement.<br><br>General guidance is provided below. Consult the submission guidelines for detailed instructions. **Make sure that all information entered here is included in the Methods section of the manuscript.**	Institutional and unit permissions were obtained. Ethical approval for this study was granted by the Committee on Human Research Publication and Ethics (CHRPE) at the Kwame Nkrumah university of Science and Technology (KNUST), Ghana with reference number CHRPE/AP/634/18. Administrative permission was also granted by district coordinating councils where aged persons lived. Individual informed consent was obtained from all aged persons. Anonymity and confidentiality were explained to participants and they were assured that withdrawal will not in any way attract sanctions. Participants were given informed consent form to fill out and sign. Participants were identified with codes to maintain confidentiality and anonymity. Researchers made sure that this study did not cause any physical, psychological or emotional harm to any participant.

Powered by Editorial Manager® and ProduXion Manager® from Aries Systems Corporation

**Format for specific study types**<br><br>**Human Subject Research (involving human participants and/or tissue)**<br>• Give the name of the institutional review board or ethics committee that approved the study<br>• Include the approval number and/or a statement indicating approval of this research<br>• Indicate the form of consent obtained (written/oral) or the reason that consent was not obtained (e.g. the data were analyzed anonymously)<br><br>**Animal Research (involving vertebrate animals, embryos or tissues)**<br>• Provide the name of the Institutional Animal Care and Use Committee (IACUC) or other relevant ethics board that reviewed the study protocol, and indicate whether they approved this research or granted a formal waiver of ethical approval<br>• Include an approval number if one was obtained<br>• If the study involved *non-human primates*, add *additional details* about animal welfare and steps taken to ameliorate suffering<br>• If anesthesia, euthanasia, or any kind of animal sacrifice is part of the study, include briefly which substances and/or methods were applied<br><br>**Field Research**<br><br>Include the following details if this study involves the collection of plant, animal, or other materials from a natural setting:<br>• Field permit number<br>• Name of the institution or relevant body that granted permission	
**Data Availability**<br><br>Authors are required to make all data underlying the findings described fully available, without restriction, and from the time of publication. PLOS allows rare exceptions to address legal and ethical concerns. See the PLOS Data Policy and FAQ for detailed information.	No - some restrictions will apply

Powered by Editorial Manager® and ProduXion Manager® from Aries Systems Corporation

A Data Availability Statement describing where the data can be found is required at submission. Your answers to this question constitute the Data Availability Statement and **will be published in the article**, if accepted.<br><br>**Important:** Stating 'data available on request from the author' is not sufficient. If your data are only available upon request, select 'No' for the first question and explain your exceptional situation in the text box.<br><br>Do the authors confirm that all data underlying the findings described in their manuscript are fully available without restriction?	
**Describe where the data may be found in full sentences. If you are copying our sample text, replace any instances of XXX with the appropriate details.**<br><br>• If the data are **held or will be held in a public repository**, include URLs, accession numbers or DOIs. If this information will only be available after acceptance, indicate this by ticking the box below. For example: *All XXX files are available from the XXX database (accession number(s) XXX, XXX.*).<br>• If the data are all contained **within the manuscript and/or Supporting Information files**, enter the following: *All relevant data are within the manuscript and its Supporting Information files.*<br>• If neither of these applies but you are able to provide **details of access elsewhere**, with or without limitations, please do so. For example:<br><br>*Data cannot be shared publicly because of [XXX]. Data are available from the XXX Institutional Data Access / Ethics Committee (contact via XXX) for researchers who meet the criteria for access to confidential data.*<br><br>*The data underlying the results presented in the study are available from (include the name of the third party*	The data are available from corresponding author only on reasonable request. This is due to the sensitive nature of the data.

and contact information or URL).<br>• This text is appropriate if the data are owned by a third party and authors do not have permission to share the data.<br><br>* typeset	
Additional data availability information:	

Powered by Editorial Manager® and ProduXion Manager® from Aries Systems Corporation

Manuscript Click here to download Manuscript manu with title page.docx

Older people's challenges and expectations of nursing care in Ghana: A qualitative study

Confidence Alorse Atakro[1] confidenceatakro@gmail.com

Abigail Atakro[2] abigaildelali29@gmail.com

Janet Sintim Aboagye[1] janetsintima@yahoo.com

Alice Aluwah Blay[1] aablay@csuc.edu.gh

Stella Boatemaa Addo[1] sbaddo@csuc.edu.gh

Dorcas Frempomaa Agyare[3] dorcas.agyare@ucc.edu.gh

Peter Adatara[4] awakape@yahoo.com

Kwaku Gyimah Amoa-Gyarteng[5] gyartengkwaku@gmail.com

Awube Menlah[6] amenlah@yahoo.com

Isabella Garti[6] darkoisabella@gmail.com

George Sedinam Boni[7] sedi.boni24@gmail.com

Osei Kwaku Berchie[1] berchieoseikwaku@gmail.com

Isaac Kwadwo Ansong[6] ikansong@vvu.edu.gh

[1]School of Nursing and Midwifery, Christian Service University College, Kumasi, Ghana

[2]Tema General Hospital, Ghana

[2]University of Cape Coast, Cape Coast, Ghana.

[4]School of Nursing and Midwifery, University of Health and Allied Sciences, Ho, Ghana

[5]Suntreso Government Hospital, Kumasi, Ghana.

[6]Valley View University, Ghana

[7]Volta Regional Hospital, Ho, Ghana

Corresponding Author

Confidence Alorse Atakro

School of Nursing and Midwifery, Christian Service University College, Kumasi, Ghana

confidenceatakro@gmail.com

Abstract

Background

Elderly persons across the globe are increasingly becoming marginalised and isolated. The population of elderly persons in Ghana is expected to increase from 4.92% in 1960 to 6.34% in 2020. The increase in the number of elderly persons has not had a corresponding increase in social and health care support systems for the elderly. There is limited evidence of the nursing care challenges and expectations by elderly persons in Ghana. This study aimed at exploring nursing care challenges and expectations of elderly persons to inform policy that could lead to improved quality of life for elderly persons in Ghana.

Materials and Methods

Qualitative exploratory descriptive study design was used in conducting this study. Semi-structured interviews were used in collecting data from 30 participants from three regions in Ghana (10 from each region). Data analysis was carried out through content analysis.

Results

Five themes were extracted from data. These themes were: 1.Inadequate information from nurses. 2. Queuing frustrations. 3. Financial burden. 4. A cry for cost subsidisation. 5. Focused elderly care demand.

Conclusion

The elderly in Ghana experience challenges of nursing care which include inadequate information, queuing frustrations and financial burdens. Elderly persons also have expectations of care which include subsidisation of cost and attention to their care needs by nurses. Many of these challenges and expectations cannot be addressed by nurses alone. There is a need for collaborative approach as well as advocacy, curricula modifications and workshops for practicing nurses on improved elderly care in Ghana.

Keywords: Elderly, advocacy, expectations, challenges, nursing, district, hospital, Ghana.

Background

The world's population of elderly persons over 60 years will nearly double from 12% to 22% between 2015 and 2050 [1], 2015a) with 80% expected to reside in low and middle-income countries (LMICs) by 2050 [2]. Elderly persons globally suffer from various health problems such as chronic conditions, injuries, depression from loneliness, malnutrition, visual problems, hearing loss and complex dental problems [3,4,5]. However, elderly persons across the globe are increasingly becoming isolated and marginalized despite the many challenges they face [6]. These challenges have been found to be proportionately higher in developing and LMICs because majority of these elderly persons are socially and economically disadvantaged [7, 8]. The higher proportion of elderly care challenges in LMICs is due to wide global variations of inequalities related to quality of life of elderly persons [9]. Many middle income and developing countries remain under-prepared to care for the high population of elderly persons [1]. In contrast to developed nations, several parts of the developing world have the majority of their elderly persons in rural areas and urban slums [10]. The response of African governments to issues of economic, health, disability, and living conditions in old age are minimal when compared to governments response on other continents [11,12, 13]. The recommended societal approach to population ageing, which includes building an age-friendly health system, requires a transformation of health systems in sub-Saharan Africa from curative models to provision of integrated care that is centered on preventive, psychosocial and cultural needs of elderly persons [1]. The developmental contributions that can be derived from elderly persons in African countries will heavily depend on the health care provided to them [14].

The number of elderly persons in Ghana is expected to increase from 4.92% in 1960 to 6.34% in 2020 [11, 14]. Population ageing is occurring at a time in which traditional systems that supported elderly persons have been affected by modernisation and globalisation resulting in downward trends of support through public welfare systems [13]. Existing inequalities in elderly care is particularly true for Ghana as two-third of her elderly population living in villages are vulnerable to greater socio-economic and health marginalisation [15]. Sub-Saharan African countries including Ghana tend to invest in health, education, and social support systems for their young populations whilst neglecting the support systems needed for elderly persons who contribute immensely to the development of their communities, families and

countries [16,17]. Though many elderly persons may eventually experience multiple health problems, being an elderly person does not imply dependence [7]. Ghana may miss the opportunity of deriving maximum benefits from elderly persons if she fails to make the appropriate adaptations and investments in these elderly persons [17]. The proportion of the elderly persons in Ghana is currently at 7.2 percent, which indicate that Ghana has one of the highest proportions of elderly persons in sub-Saharan Africa [12]. The increase in the number of elderly persons has not had a corresponding increase in social and health care support systems for the elderly [12]. Though the National Health Insurance Scheme (NHIS) of Ghana by law covers elderly persons above 70 years [18], many elderly persons above 70 years are faced with the challenge of bearing the cost of some health services such as paying for medications and investigations due to the inability of the National Health Insurance Authority (NHIA) to remit health facilities on time. Elderly persons within the age brackets of 60 and 69 have no NHIS cover although the compulsory retirement age in Ghana is 60 years and average life expectancy is 63 years [11,18]. Since nurses are the frontline of health care institutions, their role in the care of the elderly person cannot be underestimated [19]. However, a study by Oyetunde, Ojo and Ojewale found that nurses in West African countries lack the necessary knowledge for the care of the elderly [20].

Ghana currently lacks age friendly hospitals and gerontological nursing experts, though nurses are the highest number of health workers [21]. Though nurses are usually in constant contact with elderly persons at every level of health care, evidence available shows that nurses have inadequate education in gerontological nursing which subsequently leads to gaps in the care of elderly persons [20]. Inadequate knowledge in gerontology care usually results in negative health outcomes for elderly persons' [20]. Very little empirical research has been undertaken on long-term trends in psychological, cultural, health and social support systems of elderly persons in West African countries [17]. Very little is known about the health of elderly persons in Ghana. Although district hospitals are the first point of contact for many ill elderly persons in Ghana, research evidence on the challenges and expectations of elderly persons in these hospitals is seldom looked into. A report by Cohen and Menken concluded that there is an urgent need to constitute an agenda of needed research on aging in sub-Saharan Africa

including Ghana [22]. This study set out to inform Ghanaian elderly nursing care policy by identifying:

1. Challenges of elderly persons regarding nursing care in the Ghanaian health system, and
2. Expectation of elderly persons regarding nursing care in the Ghanaian health system.

Materials and Methods

Study design

Qualitative exploratory descriptive design was used in conducting this study. Qualitative exploratory descriptive design allows researchers to understand the experiences of people about a phenomenon [23]. Qualitative explorative descriptive study design therefore enabled researchers in this study to have in-depth understanding into challenges and expectations of nursing care by elderly persons in Ghana.

Study setting

Ghana is a country in West-Africa with a current projected population of 30.3 million people [24]. Ghana is divided into 10 regions. These 10 regions are divided into northern, middle and southern zones. The Northern zone consist of Upper West, Upper East and Northern regions (Ghana Health Service [25]. The middle zone consist of Brong Ahafo and Ashanti regions [25]. The southern zone is made up of Western, Central, Greater Accra, Volta and Eastern regions [25]. This study was undertaken in three purposely selected regions in Ghana: one region from the southern zone, one from the middle zone and the other from the northern zone. Volta region was selected from the Southern Zone, Ashanti region from the middle zone and the Upper West from the Northern Zone. There are 25 district hospitals in the Ashanti region, 3 district hospitals in the upper west region, and 17 district hospitals in the Volta region [25]. The bed capacities of public health hospitals in the Ashanti, Upper West and Volta are 1230, 225, and 1400 respectively [25]. The nurse patient ratio of the Ashanti, Upper West and Volta regions are 1088, 813 and 925 respectively [25]. Data was collected from these three regions because investigators wanted to have an in-depth understanding into challenges and expectation of elderly persons in many areas of Ghana.

Population and sampling

The study involved elderly persons in three regions of Ghana. A purposive sampling technique was used in selecting one region from each of the three zones. A purposive sampling technique was utilised in selecting regions because the researchers wanted to include a region from each of the three regional zones (northern zone, middle zone and southern zone) in Ghana. The Ashanti, Volta and Upper West regions were selected from each zone. Participants in this regions were also selected through a purposive sampling technique. The targeted population for this research were elderly persons 60 years and above who had visited district hospitals in selected regions. Ten participants were interviewed in each region. All participants were within the ages of 60 and 89 years. Only elderly persons who had visited the hospital during the year were involved in study.

Data collection

All participants were 60 years and above. Only elderly persons who visited district hospitals for treatments within the year of this study were included. All participants could either speak English, Ewe, Twi or wala. Ewe, Twi and wala are local languages spoken in the regions where data was collected. There was no need for a translator since members of the data collection team could speak any of the three languages. Data were collected with a semi-structured interview guide which was formulated by the research team. Interview questions were developed by the research team headed by two nursing education experts. Interview guide was pretested to identify ambiguous questions. Interviews were conducted in Twi, Ewe and wala for participants who did not speak English. Questions asked during interviews included the following: 1. Can you describe any challenges from nurses that you faced when you visited a district hospital here for treatment? 2. Describe your perception of nursing care received during your visit to the district hospital. 3. Describe how you see the current care of the elderly in Ghanaian hospitals by nurses. 4. Describe ways you think nurses can care for you better when you visit the hospital for care. Probes were used to elicit further descriptions of challenges and expectations. Data were collected within a three month period from January to March 2018. Each interview lasted between one and two hours. Data saturation was reached after interviewing 30 participants. Transcribed data were stored with a password. Folders containing data transcriptions were kept on a pen drive solely meant for the purposes of the study and kept under lock and key.

Data Analysis

Data analysis was conducted with qualitative content analysis. Data was analysed manually by research team which was headed by a professor of nursing. Holloway and Wheeler's data analysis pattern was used during data analysis [26]. This pattern takes the following form; validating, transcribing, cleaning and coding data, creating family of codes, and forming themes from family of codes [26]. The research team sat together and read through the content of field data. Data was cleaned by removing all identifiable information. Codes were found during readings of transcripts. Codes were discussed within the research team. Similar codes were used by the team to create families and similar families grouped together as themes. The themes were discussed among all members of the research team for agreement on the themes. The themes were also discussed with some participants to find out if they represented their views.

Rigour

A pretest of semi-structured interview was carried out in the Central Region. Pretest ensured that ambiguous questions were modified to make them clearer for participants. The researchers had prolonged interactions with elderly persons to ensure in-depth understanding of findings that emerged. Member checking was carried out to validate data from the participants. Data transcriptions and coding were done by the research team to ensure that the right challenges and expectations were reported. Researchers went back to elderly persons to find out if themes formulated represented their opinions.

Ethical consideration

Ethical approval for this study was granted by the Committee on Human Research Publication and Ethics (CHRPE) at the Kwame Nkrumah university of Science and Technology (KNUST), Ghana with reference number CHRPE/AP/634/18. Permissions were also sought from district assemblies where data were collected. Anonymity and confidentiality were explained to participants. Participants were assured that withdrawal from study will not in any way attract sanctions. Informed consent forms were filled and signed by participants. Participants were identified with codes to ensure anonymity. Researchers made sure that this study did not cause any physical or psychological harm to any participant.

3. Results

Demographic characteristics of participants

Majority of participants (60%) were women. All elderly persons interviewed were within the ages of 60 and 89 with majority (58.3%) falling within 60 to 70 age bracket. Sixty-six percent of elderly persons interviewed were Christians. As many as 40% of participants were widows/widowers whilst 33% were still married. Sixty seven percent of participants had primary, junior high and senior high education whilst only 33% had tertiary education. Sixty seven percent were retired whist 33% were still actively employed.

Themes

Five themes were formulated from content analysis. The themes that represented challenges and expectations of nursing care by elderly persons in Ghana were: 1. Inadequate information from nurses. 2. Queuing frustrations. 3. Financial burden. 4. A cry for cost subsidisation. 5. Focused elderly care demand.

Inadequate information from nurses

Majority of elderly persons pointed out that inadequate information from nurses in the out-patient department and other departments in Ghanaian district hospitals made them wander most of the time in the hospital environment. Many elderly persons did not have detailed explanations of nursing activities when they visited district hospitals for care. Some elderly persons attributed the lack of information from nurses to inadequate time and workload on the part of nurses. Elderly persons were of the view that nurses were usually too busy to talk with them:

> *Nurses did not explain issues into details when taking care of me. Most of the time they will come and say, Maame I want to do this and that for you. May be it is because they don't have time. Not much explanation is given on the needs and how important that task will affect my health. We also need more information on how to prevent diseases from getting to us. I think more explanations will be good. I will be happy if that can be done. I will always feel very comfortable with that part when they take their time to explain issues to us. I really think it is the lack of time in the hospital and workload [AP3].*

Inadequate information made elderly persons wander within hospital environments in a bid to find various departments within the hospital for services. Elderly persons were of the view that they should not be made to wander like younger clients:

> *Not enough information is given. Nurses will usually tell you to go and do this and that investigation but sometimes we don't even know where those places are. You have to roam around the whole place sometimes before finding places for these services. Some of the places are big and it is not easy just going round and round like that. You know, we are not that young anymore. I don't also want to blame nurses. May be their workload is too much [AP15].*

Some elderly persons thought there should be receptions in district hospitals similar to what can be found in many organisations in Ghana. They wished they could be given necessary information at these proposed receptions without having to ask for such information from nurses:

> *Sometimes you don't even know who to ask what? Nurses and other people just moving around and you need to be asking a lot of questions to know where to go. If you don't ask, mostly the staff will not really mind you. It is not easy as an elderly person to go to the hospitals and be walking round the whole place. I wish they could give enough information without asking. If they can have receptions to give us information in the hospital, it will be good [AP5].*

> *The issue for me is the information in our hospitals. Some workers should be there to explain things to us to understand well. Information is necessary to know what to do in the hospital environment. It is not very nice to be sent around any how in the hospital when you don't even have adequate information. There should be people giving us information in the hospital. Some nurses can do that for us or some people as receptionists. They should have special places for information that can help us whilst in the hospital [AP30].*

Queuing frustrations

Majority of elderly persons stated their frustrations in joining long queues for treatment in Ghanaian district hospitals. Many of the elderly persons felt that they should be given special priority because of their age and not join the regular long queues found in many Ghanaian district hospitals:

> *I am always joining long queues with the young and energetic people. There are few nurses attending to both the elderly and the young ones. They keep us in a long queue, keep us waiting before later seeing the doctor. This is frustrating. Looking at my age and I join a queue with the young ones, there is a problem. The nurses and managers of our hospitals should take a look at the situation of waiting time for us. Something should be done about it especially for us the elderly [AP28]*

> *My son look at me and my age and joining all those long queues in the hospital for treatment. Sometimes I wish I had an option to find some other treatment somewhere else, but I don't. My drugs can only be collected from here. I go and sit in the hospital sometimes from morning to evening. It is like first come first serve. The nurses don't really consider age or how old you are. I think it should be considered [AP3].*

The long waiting hours in queues have resulted in some elderly persons seeking to treat themselves with over the counter medications instead of reporting to the hospitals for treatment:

> *It is not easy when you think of going to the hospital. All that comes to mind is the long queues that will be waiting for you when you get there. Sometimes I just go to the drug store or the local chemist shop to get something for myself instead. But I think it is not the best [AP3].*

Elderly persons indicated that they usually try other sources of treatment locally because of the frustrations they experience in hospitals. However, they visit hospitals when there are complications in their conditions:

> *I spend too much time at the hospital before receiving treatment. I think nurses can let us see the doctors first. I prefer to take some drugs at home rather than to waste my time at the hospital. But when it becomes very serious, I have to be in the hospital [AP20].*

> *To be honest with you, when I get there, nurses just make me join all these long lines of people. Sometimes I feel pity for myself in such situations. Sometimes I just buy some local medicines and take to help myself rather than going to the hospital. But when the local medicines are not helping, I go to the hospital [AP1].*

Financial Burden

Many elderly persons interviewed reported that there were high financial burdens in seeking medical treatment in Ghanaian district hospitals. Though Ghana has an insurance system for the elderly persons, many of the elderly interviewed stated they had to buy most of the expensive medications that were prescribed in the hospitals and paid for some investigations:

> *Though we have insurance, we still pay for service when we go to the hospital. Many of the good medications are never on the insurance. Even some of the investigations that we are supposed to do in the hospital are not free. This puts extra financial burdens on us as elderly persons. The insurance should cover everything [AP4].*

> *They say we have insurance covering elderly persons in Ghana but I think it is not effective at all. When I go to the hospital they tell me that I have to still pay for some stuff such as investigations and medication. The insurance seems to be for only folder. I don't think this should be the case [AP2].*

Many elderly persons were financially constrained as a result of retirements from their formal jobs:

> *I am now retired and so I don't have money as I use to have. And even medication prescriptions are given to us or our relatives to go and buy for us. So the insurance is not that effective in Ghana. The government has to improve the insurance especially for elderly persons since our income has reduced [AP 7].*

Some elderly persons who were between the ages of 60 and 70 indicated their frustration at their exclusion from the health insurance for the aged in Ghana.

> *We are always paying monies on hospitalisation. The health insurance is supposed to cater for elderly persons over 70 years. What about those of us who are 65. We retire at 60 in Ghana, but the insurance starts from 70. This I don't understand. Something could be done about this for us [AP29].*

Some elderly persons were of the view that the Nation Health Insurance Authority in Ghana does not reimburse hospitals regularly and this was partly the cause for having to pay for healthcare cost even though some of them have the health insurance.

> *The health insurance people don't pay the hospitals, so they don't also give us the free service they are supposed to provide for us. The last time I went to the hospitals, the nurses gave me a long list of prescriptions which I had to buy from outside the hospitals. The hospital did not have those medications in their pharmacy so I was forced to buy them in town. The insurance should work better [AP9].*

A cry for cost subsidisation

Majority of elderly persons indicated that the cost of their care in Ghanaian district hospitals was high. Despite the introduction of a national health insurance scheme in Ghana, elderly persons still pay various amounts of money when they visit health facilities in Ghana for

treatment. Elderly persons proposed a reduction to their health care cost by the Government of Ghana:

> *There should also be a reduction in the cost involved in our care since we don't work at this age. As for this one we are pleading. We buy too many things and drugs although the government says we are catered for by the national health insurance. The last time I was there, I had to go buy some medications myself and that had to take away all of my money I was managing. This issue should be looked at for us by government. We beg them [AP7].*

> *Politicians talk on the radios and televisions and say there is health insurance for elderly persons in Ghana but in reality, we still pay when we go to the hospital. Many of the medications they write for us are always expensive ones and we always need to buy. I think we should be attended to without all the cost that we bear in district hospitals. The Government can make this happen. We are pleading for this [AP17].*

Elderly persons were of the opinions that nurses could help advocate for reduction in healthcare cost in Ghanaian hospitals:

> *I think the nurses have seen our plight and they are in the best position to talk for us. The monies we pay are too much and they know. They should help communicate this to the government for a reduction. We are pleading [AP19].*

Apart from calling on government to reduce cost for elderly person's healthcare, majority of elderly persons also said managers and nurses at the hospital level should find a way of reducing their healthcare cost:

> *I usually pay when I go to the hospital even though I have health insurance as an elderly person. Our nurses and hospital managers should try and treat us without collecting money from us. If the politicians can't help, nurses and hospital mangers should look at our situation and help. We are elderly persons and on pensions. We don't have much*

money at this stage of our lives and so we should not be paying those monies. We are pleading for this [AP29].

I feel the nurses and other workers see what we go through paying all these monies in the hospitals. They should help us. I know they can do something [AP19].

Focused elderly care demand

The theme of focused elderly care demand has two sub-themes: 1. Need for routine check-ups. 2. A call for dedicated elderly care units and consulting rooms.

Need for routine check-ups

Many elderly persons were of the view that many of the problems they faced could be reduced to the barest minimum through regular check-ups by nurses. Participants indicated that these check-ups could take place in their communities or homes. Check-ups in their homes or communities will help reduce the cost of transportation to hospitals for health care or check-ups.:

Special care should be given to the elderly person. For me, there should be regular check-up programs for us in our homes. These check-ups could be free so that many of our challenges can be prevented. It will prevent our travels and monies spent on travelling all the way to the hospitals [AP24].

Regular check-ups for the elderly person will help a lot to reduce our burden. I think sometimes these check-ups can be done in our communities. Sometimes nurses can come and see how we are doing in our homes. It will be good for us so we do not take cars and spend our little monies going to the hospital [AP26].

Some elderly persons also recommended that in the absence of the regular visits by nurses, nurses can alternatively call them on the phone on regular basis to check up on them:

> *Even if nurses can't come to us regularly, they should call us on the phone on some regular basis to find out how we are doing. We have phones now so we are ready for nurses to call us and check up on us. It will help us a lot. We will not have to think about coming all the way to the hospital for check-ups [AP7].*

> *Check-ups can even be done through calls to us by nurses so we know whether we really need to come to the hospital. I am reluctant to take a car all the way to the hospitals for check-up although I know check-ups are good for my health and I know it can prevent serious diseases. They can just call us and talk to us [AP3].*

> *Now we all have phones. Nurses can call us and we can have discussions on our health. It will be nice. Because when I think of the difficulties I will go through when I get to the hospital, I just decide to stay at home. It is not that check-ups are not good. I know that check-ups and reviews are good but there is stress when you go to the hospital for such purposes [AP8].*

Many of the elderly persons thought that regular check-ups could also be organised for them as pertains to monthly antenatal programs for pregnant women in Ghanaian hospitals.

> *Just like what is done for pregnant women, we can also have dedicated days to come for our check-up or reviews. Nurses have done it for pregnant women so why can't they do it for us too? The district hospital authorities and nurses can set a day aside in a month for all elderly persons to come for check-ups. They should publicise these check-up days for the elderly so that we know when exactly we have to come. In that case we know the day is for us and we will be attended to rapidly and quickly [AP18].*

A call for dedicated units and consulting rooms

Many elderly persons suggested that Ghanaian district hospitals should be designed to prevent queuing for elderly persons. Creation of elderly wards and consulting rooms were suggestions and expectations of the elderly to prevent queuing for the elderly in Ghanaian hospitals. Elderly persons expected nurses to dedicate a consulting room to them to reduce their waiting time at the out-patient department:

> *I mean there should be departments and nurses for elderly persons. The nurses can find a separate room or unit for us where they will see only elderly persons. These nurses should only be working to help us treat our diseases and care for other problems that we may have. This will help us elderly persons a lot. It is better than coming here and spending so much time before seeing a doctor [AP11].*

> *For us not to queue for longer period, nurses should be employed to care for elderly persons. In that case, we will just go straight to see our nurses and doctors when we go to the hospital instead of joining long queues to see the medical officer [AP9].*

Elderly persons were of the view that hospital mangers and nurses could help in setting up these special units and consulting rooms for the elderly patient:

> *Managers and nurses of the district hospitals in this country should come together and develop a plan that will make sure that there are units in every hospital just for the elderly. We will be happy if they can do this for us. When there are separated units and consulting rooms for us, I believe we will not go through any frustrations at all when we go to the hospitals [AP3].*

> *Our hospitals should create units and consulting rooms just for us to come and see specialist doctors and nurses. The managers and the nurses of the hospital should plan this for us. It will be of benefits to us. I personally don't like joining all those long lines in the hospital for treatment. It is frustrating [AP24].*

The nurses sometimes try their best but I think when they mix us elderly persons with everyone else, then it becomes frustrating for us. The hospital managers can help the nurses create special units for us. It will help if that could be considered as a matter of urgency [AP5].

Discussion

This study showed that elderly persons in Ghana have challenges and expectations of nursing care in Ghanaian district hospitals. Majority of elderly persons narrated various challenges of nursing care within Ghanaian hospitals. These challenges included inadequate information within hospitals, frustrations of queuing and financial burdens. Elderly persons who were mostly between the ages of 60 and 89 also had expectations of nursing care. These expectations included a call for a well-planned check-up system, dedicated units and consulting rooms. Elderly persons in this study also wished for cost subsidisation of their health care. Authors did not find previous studies in Ghana that explored challenges and expectations of elderly persons regarding nursing care in Ghanaian hospitals. However, in developed countries such as Australia and New Zealand where elderly care systems are available, researchers found that some elderly-care staff are non-professional workers [27, 28]. These groups of workers may have limited education for elderly care [28].

Although Registered General Nurses (RGNs) constitute the majority of health professionals in Ghanaian hospitals, they are not assessed in gerontological nursing care during their licensing examinations [29]. The inclusion of gerontological nursing in nursing schools' curricula is also optional for universities that train undergraduate nurses in Ghana. The Nursing and Midwifery Council (NMC) of Ghana could collaborate with academics to develop a gerontological nursing curriculum for nursing schools in Ghana. These ideas are supported by Aberdeen and Angus [30] who recommended leadership training in gerontology and curriculum modifications as necessary steps for achieving positive outcomes in elderly care. Gerontological nursing education and leadership training in gerontology will help deal with the challenges of nursing care experienced by elderly persons in this study. Clinical leadership in elderly care improves quality of healthcare for elderly persons [31]. Organisational collaborations and public advocacy from nurse managers in Ghanaian hospitals can contribute immensely to system changes and improve elderly care in

Ghana. Organisational collaborations and public advocacy is crucial because curriculum modification and leadership training alone cannot solve challenges of inadequate information, queuing and high financial burdens that confront the elderly in Ghanaian district hospitals. The policies for elderly persons in Ghana should be backed by favourable laws. The national aging policy [32] which sought to address many challenges of the elderly in Ghana should be included in curricula of nursing schools. The inclusion of the aging policy in nursing school curricula may be a good start towards its implementation. Clinical leaders should develop clear achievable goals for gerontological care in Ghana. These goals could include training gerontological medical and nursing specialists in other countries that have developed gerontology care systems. These gerontology specialist should return to work in Ghanaian hospitals and also teach students in gerontological care. Nursing academics and clinical nurse specialists could help establish an advanced nurse practitioner course in elderly care in Ghana. Gerontological units could later be established in health facilities to prevent challenges such as inadequate information and queuing that confront the elderly. Additional governmental policies such as modifications to the current elderly health insurance package, elderly work modalities and innovative elderly hospital services could be implemented to improve the quality of life of older Ghanaians.

Currently the NHIS only caters for elderly persons above 70 years [18] whilst life expectancy of the Ghanaian elderly person is 63 years and compulsory retirement is 60 years [33]. The national health insurance scheme should be extended to elderly persons between 60 years and 69 years since the current evidence shows that many of them may not live to 70 years to benefit from the current elderly health insurance package. Today's generation owe it as a duty to honour and guarantee better living conditions for our elderly persons [18] because elderly persons in Ghana have contributed their quota to the development of the nation [32]. The Nation Ghana must make the effort to provide elderly persons with efficient health care services and conducive living environment to ensure that they age actively and with adequate security and dignity [18]. Expectations of routine check-ups, dedicated elderly units and cost subsidisation should be considered by all stakeholders and health institutions in Ghana. A similar check-up system similar to antenatal check-ups could be planned for the elderly in Ghana to prevent many of the diseases that confront them during these periods of aging. Frequent telephone calls could be placed to elderly persons to find out their challenges. Ageing could also be defined in terms of functionality

rather than a stage in a life time because some elderly persons could function at the age of 60 and others may retire earlier [34,35]. The United Nation's Organisation (UNO) recommends rehiring some experienced retired persons to enhance knowledge transfer to younger workforce at work places [36]. The UNO recommends development of regulations for the utilisation of these elderly persons who are over 60 years [36]. Retiring every elderly persons at the age of 60 may be disadvantageous to institutions who could benefit from their experiences. Allowing the abled elderly persons to work beyond the mandatory retirement age will also make it possible for them to pay for care and prevent the demand for cost subsidisations. Focused elderly care could include educating the elderly to engage in various forms of activities for income and health. Evidence available shows that social activities by elderly persons improves their quality of life [37]. These activities may change for some elderly persons from previous roles of work and caring for own children to activities such as volunteering and grand-parenting [36]. Another way of doing away with the many challenges faced by the elderly in Ghanaian district hospitals is to introduce aged care home services as done in other countries [27, 28]. The introduction of elderly homes in developed countries have not been without challenges. Evidence available shows that increasing commercialisation, lack of regulation and inadequate monitoring can result in poor quality care of elderly persons in aged homes [28]. Therefore, any introduction of aged home care services in Ghana must be done with the appropriate regulatory and monitoring mechanisms. This regulatory mechanism could include the use of professionally trained persons in these elderly care homes and adequate remuneration of these professionals. Patients and their relatives in these homes should be actively involved in care with their concerns and opinions considered during care. Elderly persons have the right to choose the kind of health service they want. Elderly care models that can be implemented in Ghana should integrate client choices, community/primary health care services, residential and non-residential elderly care services [38]. In the use of this models, client assessment should be done by social welfare personnel after the application for a service by an elderly person [27]. This model has been found to work in some countries in caring for elderly persons [38].

Strengths of the study

This study discussed challenges and expectation of the elderly regarding nursing care in Ghana. It is one of the few studies that explored these phenomenon in Ghana. The researchers also covered the three zones in Ghana by taking data from a district in the northern, middle and southern zones. A rigorous result was ensured by including nursing education experts in this study. This study has discussed models of elderly care as pertains to other continents and recommended elderly care models for Ghana. Further education for nurses and medical officers in gerontology has been suggested in a bid to improve expertise of gerontology care in Ghana.

Limitations of Study

Though generalisation is not entirely impossible in qualitative research, a further quantitative study covering many more regions in Ghana would help provide a more general perspective. Data from three regions in Ghana may not be sufficient to make adequate generalisation from study results. This study only looked at district hospitals which are public hospitals in Ghana. Further studies covering private, regional and tertiary health institutions will also be necessary in the future to compare results to that of district hospitals. Generalisation of results to populations outside the study population should be done with careful considerations.

Conclusion

Elderly persons in Ghana have challenges as well as expectations of nursing care. Challenges include inadequate information, queuing, and financial burdens. Expectation of nursing care included organisation of regular routine check-ups, dedication of a ward to the elderly and cost subsidisation of elderly care. Though nurses cannot address all the challenges of elderly persons in Ghanaian district hospitals, they can advocate for better care for elderly persons in Ghana. An advanced nurse practitioner programme can be developed by Ghanaian nurse academicians and clinical nurse specialists. Bottom top approaches should be utilised in elderly care where concerns and opinions of the elderly and their relatives are considered in their care. Curricula frameworks for teaching elderly care in Ghana should be formulated through further research. The current aged policy could be included in nursing curricula to create some awareness of elderly care in Ghanaian nursing students. Workshops should also be organised for practicing nurses and nursing leaders on the care of the elderly. Elderly persons could be retired based on

functionality rather than chronological age. This will make it possible for many institutions to benefit from their knowledge and experience. Rehiring active elderly persons may also help them afford necessary health care in Ghana.

Acknowledgements
The authors are grateful to aged persons who participated in this study.

Authors' contributions
CAA conceptualised, designed study, collected data, analysed data and prepared manuscript. AA designed, collected and analysed data. JSA conceptualised, designed, collected and analysed data. SBA conceptualised, designed, collected and analysed data. DFA conceptualised, designed, collected and revised manuscript. PA conceptualised, designed, analysed and revised the manuscript. KGA conceptualised, designed, collected and analysed data. AM conceptualised, designed, collected and analysed data. IG conceptualised, designed, collected and analysed data. GSB conceptualised, collected and analysed data. OKB conceptualised, designed, collected and analysed data. IKA conceptualised, designed, collected and analysed data. All authors read and approved the final manuscript.

Data availability statement
The data are available from corresponding author only on reasonable request. This is to protect and maintain participants' anonymity and confidentiality.

Reference

1. World Health Organisation [WHO]. *World Report on Ageing and Health*. Geneva: WHO; 2015a. Available from: https://www.who.int/ageing/events/world-report-2015-launch/en/

2. United Nations Organisation [UNO]. *World population ageing*. New York: United Nations; 2017. Available from: https://www.un.org/en/development/desa/population/publications/pdf/ageing/WPA2017_Highlights.pdf

3. Kohler IV, Payne CF, Bandawe, C, Kohler H. The Demography of mental health among mature adults in a low-Income, high-HIV-prevalence context. *Demogr*, 2017; 54(4): 1529–1558. doi:10.1007/s13524-017-0596-9

4. Tynan A, Deeth L, Mckenzie D. An integrated oral health program for rural residential aged care facilities : a mixed methods comparative study, *BMC Heal Serv Res.* 2018; 18, 515. https://doi.org/10.1186/s12913-018-3321-5

5. Chen EYH, Sluggett JK, Ilomäki J, Hilmer SN, Corlis M, Picton LJ, Dean L, Alderman CP, Farinola N, Gailer J, Grigson J, Kellie AR, Putsey PCJ, Yu S, Bell JS. Development and validation of the Medication Regimen Simplification Guide for Residential Aged. *Clin Interv in Aging*. 2018; 13: 975–986. Available from https://www.ncbi.nlm.nih.gov/pmc/articles/PMC5963487/pdf/cia-13-975.pdf

6. Hasan H, Linger H. Enhancing the wellbeing of the elderly: Social use of digital technologies in aged care. *Educ Geron.* 2016; 42(11): 749–757. https://doi.org/10.1080/03601277.2016.1205425

7. Kailembo A, Preet R, Williams, JS. Socioeconomic inequality in self-reported unmet need for oral health services in adults aged 50 years and over in China, Ghana, and India. *Int J of Equi and Heal*. 2018; 17:99. 99. https://doi.org/10.1186/s12939-018-0812-2

8. Hosseinpoor AR, Bergen N, Kostanjsek N, Kowal K, Officer A, Chatterji S. Socio-demographic patterns of disability among older adult populations of low-income and middle-income countries: results from World Health Survey. *Int J of Publ heal.* 2016; 62: 337-345. doi 10.1007/s00038-015-0742-3

9. World Health Organization [WHO]. *Promoting health and reducing health inequities by addressing the social determinants of health*. Geneva: WHO; 2010. Available from: http://www.euro.who.int/__data/assets/pdf_file/0016/141226/Brochure_promoting_health.pdf

10. De-Graft A A. Health and Health care in Ghana 1957-2017. Oxford: Oxford University Press; 2017. Available from https://www.researchgate.net/profile/Ama_De-Graft_Aikins/publication/313643435_Health_and_Healthcare_in_Ghana_1957-2017/links/5a21886b0f7e9b71dd032dfa/Health-and-Healthcare-in-Ghana-1957-2017.pdf

11. Ghana Statistical Service. *2010 Population & Housing Census Report: The Elderly in Ghana.* Ghana: Ghana Statistical Service; 2013. Available from https://new-ndpc-static1.s3.amazonaws.com/CACHES/PUBLICATIONS/2016/06/06/Asante+Akim+South+2010PHC.pdf

12. Mba CJ. Population Ageing in Ghana: Research Gaps and the Way Forward. *J of Aging Res,* 2010; 1-8. doi:10.4061/2010/672157

13. World Health Organisation [WHO]. *World report on Ageing and Health*. Geneva: WHO; 2015b. Available from: https://apps.who.int/iris/bitstream/handle/10665/186463/9789240694811_eng.pdf;jsessionid=6DCBA6417D27F92F930AAC6D70417923?sequence=1

14. Ghana National Population Council. RAPID—The Change We Seek: Population and Development. Accra: Republic of Ghana. Available from https://www.healthpolicyproject.com/pubs/154_GHANARAPIDBookletFINAL.pdf

15. Kumar V, Acanfora M, Henessey CH, Kalache A. (2001). Health Status of the Rural Elderly. *J of Rur Heal*. 2001;17(4): 328-331. Available from https://onlinelibrary.wiley.com/doi/pdf/10.1111/j.1748-0361.2001.tb00282.x

16. Agyemang FA. *Survival Strategies of the Elderly in Rural Ghana.* Doctoral Thesis. Ghana: University of Ghana; 2014. Available from https://pdfs.semanticscholar.org/40d1/d44a36cc90317ae93bf931e11672b3156c15.pdf

17. Balamurugan J, Ramathirtham G. Health Problems of Aged People. *Int J of Res in Soc Sci.* 2012; 2(3): 139-148. Available from http://citeseerx.ist.psu.edu/viewdoc/download?doi=10.1.1.415.9543&rep=rep1&type=pdf

18. National Health Insurance Scheme. Membership. Accra: National Health Insurance Authority of Ghana. Available from http://www.nhis.gov.gh/membership.aspx

19. Smeltzer SC, Bare BG, Hinkle JL & Cheever KH. *Brunner and Suddart's Textbook of Medical-Surgical Nursing (11th ed).* Lippincott William & Wilkin: Philadelphia, Pa, USA; 2008.

20. Oyetunde MO, Ojo OO, Ojewale LY. Nurses' attitude towards the care of the elderly: Implications for gerontological nursing training. *J of Nurs Educ and Pract.* 2013; 3(7): 150-156. doi: 10.5430/jnep.v3n7p150

21. De Cavalho IA, Bykes J, Acquah C, Amofah G, Biritwum R, Pannisett U, Goodwin J, Beard J. Informing evidence-based policies for ageing and health in Ghana. *Bullet Worl Heal Org.* 2015; 93: 47–51. doi: http://dx.doi.org/10.2471/BLT.14.136242

22. Cohen B, & Menken J. *Aging in Sub-Saharan Africa: Recommendations for Furthering Research.* USA: National Academy of Sciences; 2006. Available from https://www.ncbi.nlm.nih.gov/pubmed/20669443

23. Polit DF, & Beck CT. *Essentials of nursing research: Appraising Evidence for Nursing Practice*. United Kingdom: Wiley-Blackwell; 2013.

24. Ghana Statistical Service (GSS). *2018 Projected Population*. Ghana: Ghana Statistical Service; 2018. Available from: http://statsghana.gov.gh/

25. Ghana Health Service. *The health sector in Ghana: Facts and figures*. Accra: GHS; 2015. Available from: http://ghanahealthservice.org/downloads/Facts+Figures_2018.pdf

26. Holloway I, Wheeler S. *Qualitative research in nursing and healthcare*: John Wiley & Sons; 2010. Available from: http://eu.wiley.com/wileyCDA/wileyTitle/productCd1119096367.html.

27. Woods M, Phibbs S, Severinsen C. 'When morals and markets collide': Challenges to an Ethic of Care in Aged Residential Care, *Eth and Soc Wel.* 2017; 11(4): 365-381. doi: 10.1080/17496535.2017.1287210

28. McKenna H, Keeney S, Bradley M. Nurse leadership within primary care: The perceptions of community nurses, GPs, policy makers and members of the public. *J of Nurs Manag*. 2004; 12: 69–76. Available from https://onlinelibrary.wiley.com/doi/full/10.1046/j.1365-2834.2003.00419.x

29. Nursing and Midwifery Council. (2015). Revised Nursing Curriculum. Accra: Nursing and Midwifery Council.

30. Aberdeen S, Angus JA. Systematic Approach to Curricula Development for Aged-Care Leadership. *Educ Geront*. 2007; 31(6): 481-497. https://doi.org/10.1080/03601270590928198

31. Pereles L, Lockyer J, Ryan D, Davis D, Spivak B, & Robinson B. The use of the opinion leader in continuing medical education. *Med Teach*. 2003; 25(4): 438–441. doi: 10.1080/0142159031000137472

32. Government of Ghana. *National Ageing Policy*. Ghana: Ministry of Employment and Social Welfare; 2003.

33. Ghana Statistical Service. (2013). *2010 Population and Housing Census Report*: The Elderly in Ghana. Accra: Ghana Statistical Service.

34. Zacher H, & Rudolph CW. Successful aging at work and beyond: A review and critical perspective. *Adv Serie in Manag*. 2017; 17: 35–64. https://doi.org/10.1108/S1877-636120170000017004

35. Potocnik K, Sonnentag S. A longitudinal study of well-being in older workers and retirees: The role of engaging in different types of activities. *J of Occup and Org Psych*. 2013; 1-2. doi:10.1111/joop.12003

36. Fall, P. L., Terzi, C., & Joint Inspection Unit. *Use of retirees and staff retained beyond the mandatory age of separation at united*. Geneva: UNO; 2014. Available from: https://www.unjiu.org/sites/www.unjiu.org/files/jiu_document_files/products/en/reports-notes/JIU%20Products/JIU_NOTE_2014_1_English.pdf

37. National Board of Health and Welfare. Intercultural Social Work. Available from http://www.socialstyrelsen.se/Lists/Artikelkatalog/Attachments/18063/20106-9.pdf

38. Luckett, T., Phillips, J., Agar, M., Virdun, C., Green, A., & Davidson, P. M. (2014). Elements of effective palliative care models: a rapid review. BMC Health Services Research, 14(1), 1–22. https://doi.org/10.1186/1472-6963-14-136
