# Supplementary material for: Older people’s challenges and expectations of healthcare in Ghana: A qualitative study
Source: PLoS One. 2021 Jan 19;16(1):e0245451. doi: 10.1371/journal.pone.0245451 (PMC7815149; doi:10.1371/journal.pone.0245451)
Supplement: Attachment — Submitted filename: RESPONSE TO REVIEW COMMENTS.docx [file pone.0245451.s004.docx]

**RESPONSE TO REVIEW COMMENTS FROM EDITOR**

| Comments from editor | Response to review comments from editor |
| --- | --- |
| 1.When submitting your revision, we need you to address these additional requirements.  Please ensure that your manuscript meets PLOS ONE's style requirements, including those for file naming. | 1.Plos one requirements were followed in the submission of manuscript. |
| 2.We note that you have indicated that data from this study are available upon request. PLOS only allows data to be available upon request if there are legal or ethical restrictions on sharing data publicly. For information on unacceptable data access restrictions, please see http://journals.plos.org/plosone/s/data-availability#loc-unacceptable-data-access-restrictions.  In your revised cover letter, please address the following prompts:  a) If there are ethical or legal restrictions on sharing a de-identified data set, please explain them in detail (e.g., data contain potentially identifying or sensitive patient information) and who has imposed them (e.g., an ethics committee). Please also provide contact information for a data access committee, ethics committee, or other institutional body to which data requests may be sent.  b) If there are no restrictions, please upload the minimal anonymized data set necessary to replicate your study findings as either Supporting Information files or to a stable, public repository and provide us with the relevant URLs, DOIs, or accession numbers. Please see http://www.bmj.com/content/340/bmj.c181.long for guidelines on how to de-identify and prepare clinical data for publication. For a list of acceptable repositories, please see http://journals.plos.org/plosone/s/data-availability#loc-recommended-repositories.  We will update your Data Availability statement on your behalf to reflect the information you provide. | 2.Cover letter was modified to reflect data availability. An audit trail has now been provided that shows data collection at every stage and could show some challenges and expectations described by older people. Minimal data collected has also been provided to enable replication of this study. The number of the ethical committee provided for request of full data set. |
| 3. Please include additional information regarding the aemi-structured interview guide used in the study and ensure that you have provided sufficient details that others could replicate the analyses. For instance, if you developed a guide as part of this study and it is not under a copyright more restrictive than CC-BY, please include a copy, in both the original language and English, as Supporting Information. Also, pretesting of this guide was referred to in the text but further details concerning the nature of number of participants is required. Furthermore, please expand on the recruitment of participants to this study and any inclusion/exclusion criteria employed. | 3. A copy of interview guide provided. all participant could speak English and did not need any translation for researchers. Additional information on the pretest such as the number pf people pre-tested has now been added. |
| 4. Abstract  No need for prevalence figures in background information | 4. The prevalence has now been removed as suggested. |
| 5. Introduction  Make sure you justify why qualitative descriptive approaches are so valuable | 5. Justification of the qualitative study in introduction has now been included and highlighted. |
| 6. Methods  You need to consider a framework which will support your write up e.g., COREQ or SRSQ – you need this to make sure you address each requirement of these – again it is not about a correct answer but that your method make sense in terms of the results you give e.g., sample size considerations? when I started to look at this type of approach this article talks about content analysis and frameworks https://journals.sagepub.com/doi/full/10.1177/1937586715614171 | 6. The COREQ framework has now been used in reporting work. The framework of COREQ was used in reporting all sections of the study. |
| 7. Study design  You will need to give a better reference for your qualitative descriptive design e.g., a paper which gives key steps that are expected to be taken e.g., a specific paper which mentions some options could be. A general text book which is not specific to the design wouldn’t be appropriate. Reconsider this and within this consider your paradigmatic stance and methodological stance | 7. This suggestion has been complied with and highlighted. |
| 8. You need to justify your selected interview guide – did you do a cognitive interview before? Was it piloted – did you use literature to work out questions? Why so few questions? Not looking for a correct answer but your choices need justification | 8. Justification now provided for the interview guide and highlighted. Questions were developed based on literature review. A pilot was done with 10 older people and ambiguous questions were modified. |
| 9. You will need to include a full audit trail without this it will be hard to follow how you got the results that you have obtained - without this the paper will be rejected at the next point. | 9. The audit trail of this study has now been provided. |
| 10. Does your data analysis approach fit with the descriptive design you mention above? Please justify your choices | 10. This suggestion has been complied with and highlighted |
| 11. Results  I would like to see themes at the minor level from an audit trail | 11. Audit trail now provided. |
