# Supplementary material for: Older people’s challenges and expectations of healthcare in Ghana: A qualitative study
Source: PLoS One. 2021 Jan 19;16(1):e0245451. doi: 10.1371/journal.pone.0245451 (PMC7815149; doi:10.1371/journal.pone.0245451)
Supplement: Attachment — Submitted filename: older people paper.docx [file pone.0245451.s006.docx]

**Title**: I suggest that the word “***nursing care”*** in the title be replaced with “***healthcare”*** as findings from the study were not exclusively nursing care issues

**Abstract**

The abstract was well structured and well written, but minor revisions required. See main document.

**Background**

The background was well written to set the stage for the study. Very minor revisions required.

**Methods**

This section although it read well, there were few repetitions. Some revisions required.

**Findings**

The findings section was well structured for easy read, however, few observations made and outlined as follows:

1. The descriptions of the elderly demographic characteristic did not play any role in the study as it would have been interesting elaborating on that. For example, it was reported that 33% of the older people who were presumed to have been retired were still in active employment. The detail of this was missing. Also, how it impacted their health and medical cost was also left hanging. Furthermore, majority of the older people were females. It would have been interesting to explore and report on the motivation for health seeking behaviour among the two gender and whether marital status and number of children played a role in their healthcare needs and hospital attendance.
2. The descriptions of the codes (variables) in the themes well not detailed enough. Moreso, most of the themes described were basic healthcare delivery or system challenges and not exclusive nursing care issues. There were some overlaps, for example, the themes *financial burden* and *a cry for cost subsidisation* read pretty much the same and could be collapsed as one theme. I believe repetitions and discrepancies will be minimised if further descriptions are given to all the codes/variables. The lengthy quotations should be reduced.

**Discussion**

The discussion needs a major revision. The discussion as it is shows a substantial difference of what is discussed and what was reported in the findings. For example, the chunk proportion of information on nursing education in gerontology and licencing examination in that field has no relation to the findings. The study did not look at the courses nurses read in training and therefore has no business discussing that. There is no consistency in the discussion as some of the information given was out of context.

The study did not look at health service organisation and implications on the care of the elderly, yet, the discussions covers it. I suggest that the discussion section should be rewritten and attention paid to the findings of the study.

**Limitation**

Well written

**Recommendations**

No recommendations suggested

**Referencing**

The recommended referencing style and format must be adhered to.

**Please see main document for further comments.**
