# Supplementary material for: Older people’s challenges and expectations of healthcare in Ghana: A qualitative study
Source: PLoS One. 2021 Jan 19;16(1):e0245451. doi: 10.1371/journal.pone.0245451 (PMC7815149; doi:10.1371/journal.pone.0245451)
Supplement: Attachment — Submitted filename: Responses to reviewers17thmay20.docx [file pone.0245451.s007.docx]

**RESPONSE TO REVIEWERS**

**Response to reviewer 1**

| 1. Study setting Page 5 under settings: lines 2 -5, the authors stated that Ghana has 10 regions and listed the 10 regions. Now Ghana has 16 Regions. I recommend that the 16 regions should be used since the work is yet to be published. When stating the regions, for instance Ashanti Region, the R for the region should be an upper case. For example, Volta Region, Ashanti Region etc. | 1.These recommended corrections have been adhered to and highlighted in manuscript |
| --- | --- |
| 2.Data collection  Page 6, line 2, I suggest that the authors use researchers instead of investigators. | 2.This suggestion had been adhered to and highlighted. |
| 3. Page 6, line 4: the authors stated that the interview guide was tested on 10 elderly persons; were these 10 elderly persons selected from the 3 research settings or other settings? Please state it. | 3. The pretest was conducted in another region (Central region). This is now stated in manuscript as suggested. |
| 4. Pages 6 and 7 seem to have repetitions:  • lines 3-6, the authors stated that “The interview guide was developed based on literature and pre-tested on 10 elderly persons in order to identify and modify ambiguous questions. Semi-structured interviews (Appendix 1) were conducted in English since all participants could speak English.”  • Last sentence on page 6 and first sentence on page 7 seems to be saying the same thing; “Interview guide was pretested on ten older people to identify ambiguous questions. Interviews were conducted in English”.  The authors should please work on the repetition. | 4. Repetitions were removed throughout manuscript. |
| 5. Rigour  Page 7 line 1 should be “a pretest of semi-structured interview guide was …” the guide was omitted. | 5. This recommended correction has been adhered to and highlighted. |
| 6. Ethical considerations  Page 8, the last sentence: the authors stated that “Researchers made sure that this study did not cause any physical or psychological harm to any participant”. Please state how these were ensured. | 6. This has now been corrected. Researchers avoided questions that could cause any form of psychological trauma. |
| 7. Demographic characteristics of participants  Page 8, the authors should be consistent with the use of the percentages. Some are in words and others are in figures. Please reconcile. | 7. This recommended suggestion has been implemented. |
| 8. Discussions  • Page 17: instead of using ‘elderly persons in this study’ authors should rather use participants. This should be effected throughout the manuscript.  • Page 19, line 20: The authors should use client instead of patient since not all aged sent to the aged homes for care are sick. | 8. This change has been done as recommended. |
| 9. Strengths of the study  Page 20 line 4: throughout the manuscript, I did not see any recommendation for medical officers to be trained in Gerontology as stated here by the authors. Please delete the medical officers if it has not earlier been stated. | 9. This correction has been done. |
| 10. There are few grammatical errors. I suggest the authors employ the services of a language editor | 10. The help of an editor was sought to make necessary grammatical corrections within manuscript. |

**Response to reviewer 3**

| 1. Title: I suggest that the word “nursing care” in the title be replaced with “healthcare” as findings from the study were not exclusively nursing care issues. | 1. This suggestion has been adhered to. |
| --- | --- |
| 2. **Abstract**  The abstract was well structured and well written, but minor revisions required. See main document. | 2. Minor correction made in abstract. |
| 3. **Background**  The background was well written to set the stage for the study. Very minor revisions required. | 3. Minor correction made in background. |
| 4. **Methods**  This section although it read well, there were few repetitions. Some revisions required. | 4. Repetitions now removed as recommended. |
| 5.The descriptions of the elderly demographic characteristic did not play any role in the study as it would have been interesting elaborating on that. For example, it was reported that 33% of the older people who were presumed to have been retired were still in active employment. The detail of this was missing. Also, how it impacted their health and medical cost was also left hanging. Furthermore, majority of the older people were females. It would have been interesting to explore and report on the motivation for health seeking behaviour among the two gender and whether marital status and number of children played a role in their healthcare needs and hospital attendance. | 5. This suggestion has been complied with and highlighted within work. However, differences in heath seeking behaviours between females and males was not explored in this study. |
| 6. The descriptions of the codes (variables) in the themes well not detailed enough. Moreso, most of the themes described were basic healthcare delivery or system challenges and not exclusive nursing care issues. There were some overlaps, for example, the themes financial burden and a cry for cost subsidisation read pretty much the same and could be collapsed as one theme. I believe repetitions and discrepancies will be minimised if further descriptions are given to all the codes/variables. The lengthy quotations should be reduced. | 6. The recommendation has been complied with. Overlapping themes have now been condensed to avoid repetitions. There is also now more concentration on health workers rather than just nurses. Lengthy quotations reduced as recommended. |
| 7. The discussion needs a major revision. The discussion as it is shows a substantial difference of what is discussed and what was reported in the findings. For example, the chunk proportion of information on nursing education in gerontology and licencing examination in that field has no relation to the findings. The study did not look at the courses nurses read in training and therefore has no business discussing that. There is no consistency in the discussion as some of the information given was out of context.  The study did not look at health service organisation and implications on the care of the elderly, yet, the discussions covers it. I suggest that the discussion section should be rewritten and attention paid to the findings of the study. | 7. The discussion has been rewritten as recommended. Discussions now organised to be in line with findings as recommended in order to stay away from extensive discussions that did not border on findings specifically. |
| 8. Referencing  The recommended referencing style and format must be adhered to. | 8. Referencing format adhered to as recommended. |
