# Supplementary material for: Older people’s challenges and expectations of healthcare in Ghana: A qualitative study
Source: PLoS One. 2021 Jan 19;16(1):e0245451. doi: 10.1371/journal.pone.0245451 (PMC7815149; doi:10.1371/journal.pone.0245451)
Supplement: Attachment — Submitted filename: RESPONSE TO REVIEWERS.docx [file pone.0245451.s008.docx]

**RESPONSE TO REVIEWERS**

RESPONSE TO REVIEWER

| **Comment** | **Response** |
| --- | --- |
| Comment 1: Under theme 1, the use of verbs such as thoughts, wish, and felt are difficult to measure. Hence, it they must be written as reported expressions. For example, … some participants shared their thoughts that...Others said that they wished…. | Response 1: The recommended change has been complied with and highlighted within manuscript. |
| Comment 2: Under theme 2, similar observations were made for example…in line 2 sentence 2 the word felt was used. | Response 2: The recommended change has been complied with and highlighted within manuscript. |
| Comment 3: Under theme 3, the second sentence should begin with the word “Although” instead of though. | Response 3: The recommended change has been complied with and highlighted within manuscript. |
| Comment 4: Under the fourth theme, subtheme 1, the check-ups should read check-up visits from health workers, not by health workers. | Response 4: The recommended change has been complied with and highlighted. |
