# Supplementary material for: Older people’s challenges and expectations of healthcare in Ghana: A qualitative study
Source: PLoS One. 2021 Jan 19;16(1):e0245451. doi: 10.1371/journal.pone.0245451 (PMC7815149; doi:10.1371/journal.pone.0245451)
Supplement: Attachment — Submitted filename: Response to reviewers15thNov20.docx [file pone.0245451.s009.docx]

**Response to reviewers**

| **Response to reviewer 3** | |
| --- | --- |
| **Comment** | **Response** |
| Comment 1:This study of perceptions of health care among a population of elderly patients addresses an important subject. However, I am concerned that the study lacks a basic hypothesis which leads to a-theoretical purposive sampling and difficulty in interpreting results. | Response 1: The authors did not formulate a hypothesis in the study. The study used a content analysis approach to explore challenges and expectations of older people in Ghanaian health system and reported this using COREQ criteria for reporting qualitative research (cited in study). The study did not utilise a grounded theory approach and therefore did not use a theoretical sampling technique. These methods were further clarified by the 4^th^ reviewer as she/he states “This research was based on content analysis, which may not be based on grounded theory. So, a theoretical foundation cannot be achieved based on the authors' methodology”. |
| Comment 2: It is unclear if saturation was achieved and if so by what criteria; rather, the approach seems to have been to select a qualitative sample size ahead of the study. | Response 2: This has now been clarified in the data collection section as follows: Five participants were initially interviewed in each region. Additional five persons were interviewed in each region as saturation was not determined with the initial interviews. Saturation was determined after interviewing the 30th participant. |
| Comment 3: The paper also does not frame the study in any larger conversations about care for aging populations or quality of care more broadly. Though local by nature, qualitative research is made valuable to various readers through this final process of synthesizing and making connections. | Response 3: The difference in the quality of healthcare received by older people in developed and developing countries has now been stated in abstract to set the tone for the details in differences in background and discussion. The first section of the background is discussed in line with global dynamics in ageing. This has now been given a broader perspective by comparing health systems for the aged in developing countries to developed countries. The difference in care is mostly due to social and economic disadvantages in African countries (this point made and highlighted). The discussion section has also now suggested some evidence-informed global practices that could be adapted in Ghana to improve the health of older Ghanaians. These changes are highlighted in the study. |
| **Response to reviewer 4** | |
| **Comment** | **Response** |
| Comment 1: In the abstract, the authors stated, "Elderly persons across the globe are increasingly becoming marginalised and isolated." This research focuses on developing countries, so the author should focus this sentence on the issues in developing countries. | Response 1: This suggestion has been made and highlighted in manuscript (abstract). |
| Comment 2: In the introduction, the authors delineate the condition of healthcare among older people broadly. The background section should include developed countries' conditions and the differences from LMICs that the authors deal with in this research for international readers. | Response 2: This suggestion has now been included. Aged care systems such as residential and community care in developed countries are now compared with developing and middle-income countries. The changes have been highlighted in the background of the study. |
| Comment 3: In the introduction, again, several grammatical mistakes such as the many challenges, very little empirical, and so on. The authors should check wording seriously in the whole manuscript. | Response 3: grammatical errors were corrected throughout the manuscript with the assistance of a professional editor. |
| Comment 4: In the sample section of the method, there is no definition of older persons. There is various definition regarding older people/patients. The authors should show the definition with a reference. | Response 4: The United Nations definition of older persons, that is persons who are 60years or over, has now been included and highlighted. |
| Comment 5: In the method section's measurement, the authors clearly described the rigor of the method. To improve the rigor, the authors can include the researchers' reflectivity, such as researchers' backgrounds. | Response 5: This suggestion has now been included and highlighted. The background of the authors as qualitative researchers further helped in ensuring qualitative rigor in the study. |
| Comment 6: The result section should include a table of the background of the participants. | Response 6: A table has now been included and highlighted. |
| Comment 7: In the limitation section of the discussion, the authors delineate the generalizability of this study. Qualitative research should be assessed regarding credibility, transferability, dependability, and confirmability. This study has a high level of truthfulness. These four criteria should be evaluated through the manuscript. | Response 7: These suggested corrections have now been done and highlighted. |
